# Novel Quaternary Ammonium Derivatives Based on Apple Pectin

**DOI:** 10.3390/polym16233352

**Published:** 2024-11-29

**Authors:** Magdalena-Cristina Stanciu, Daniela Ionita, Daniel Tȋmpu, Irina Popescu, Dana Mihaela Suflet, Florica Doroftei, Cristina G. Tuchilus

**Affiliations:** 1 “Petru Poni” Institute of Macromolecular Chemistry, 41A, Gr. Ghica-Voda Alley, 700487 Iasi, Romania; ionita.daniela@icmpp.ro (D.I.); dtimpu@icmpp.ro (D.T.); ipopescu@icmpp.ro (I.P.); dsuflet@icmpp.ro (D.M.S.); florica.doroftei@icmpp.ro (F.D.); 2 Faculty of Medicine, “Grigore T. Popa” University of Medicine and Pharmacy, 16, University Street, 700115 Iasi, Romania; ctuchilus@yahoo.com

**Keywords:** apple pectin, quats, antipathogenic activity

## Abstract

New quaternary ammonium derivatives (quats) based on apple pectin (PA) were synthesized by the chemical modification of native polysaccharides with various quaternization mixtures containing epichlorohydrin (ECH) and a tertiary amine. Pectin derivatives (QPAs) were studied by elemental analysis, conductometric titration, Fourier-transform infrared spectroscopy (FTIR), and ^13^C nuclear magnetic resonance (^13^C NMR). Viscosity measurements enabled the evaluation of the viscosity average molar mass (M_v_) for the unmodified polysaccharide, as well as its intrinsic viscosity ([η]) value. Dynamic light scattering (DLS) analysis revealed that the PA and its quats formed aggregates in an aqueous solution with either a unimodal (PA) or bimodal (QPAs) distribution. Scanning transmission electron microscopy analysis (STEM) of the PA and its derivatives demonstrated the presence of individual polymeric chains and aggregates in aqueous solution, with the smallest sizes being specific to amphiphilic polymers. Thermal stability, as well as wide-angle X-ray diffraction (WAXD) studies, generally indicated a lower thermal stability and crystallinity of the QPAs compared with those of the PA. Antipathogenic activity demonstrated that the PA and its derivatives exhibited effectiveness against *S. aureus* ATCC 25923 bacterium and *C. albicans* ATCC 10231 pathogenic yeast.

## 1. Introduction

Pectin or pectic substances are a group of narrowly connected anionic heteropolysaccharides with a high molecular weight (20–400 kDa) and are found in the primary and secondary cell walls of plants [1,2,3]. Pectic substances are biocompatible, biodegradable, and nontoxic polysaccharides. The chemical structure of pectin is heterogeneous, based on its origin, location in the plant, and extraction method. Thus, the pectin chemical structure may comprise homogalacturonan (HG) (a), rhamnogalacturonan I (RG-I) (b), and rhamnogalacturonan II (RG-II) (c) domains. (a) HG is the primary structural element of pectin, composed of galacturonic acid (GA) units, which are linearly linked by α-(l-4)-D glycosidic bonds (“smooth regions”). Chemically, the GA units of pectin are methyl-esterified on their carboxylic group (C^6^) and O-acetyl-esterified at O^2^ and/or O^3^. The methoxylation degree (DM) of pectin allow for its classification into high-methoxyl pectin (HMP) (DM > 50%) and low-methoxyl pectin (LMP) (DM < 50%). (b) RG-I is made up of linear alternate GA and rhamnose (Rha) residues (“smooth regions”). (c) RG-II is composed of a branched backbone made up of alternating GA and Rha residues, along with different side chains that include galactan, arabinan, apiose, xylose, and other complex units (“hairy regions”) [3].

Industrially, pectins are most often extracted from citrus fruit peel [4] and apple pomace [5] at a low pH and high temperature, and they are primarily homogalacturonans. Pectins are utilized in the food industry as gelling agents, texturizers, thickeners, emulsifiers, and substitutes for fat or sugar in low-calorie diets [4,5,6,7]. Pectins are also used in the compositions of pharmaceutical preparations and nutraceuticals, or as texture enhancers, paper substitutes, or food wrappings [8]. Cancer therapy [9], gene transfer [10], drug delivery [11], neuroprotective activity [12], anti-inflammatory and immune modulatory activity [13], gastroesophageal reflux/gastric ulceration [14], and anti-diarrheal treatment [15] are among the most common medical applications characteristic of pectin-based biomaterials. Pectins have a high ability to form gels. So, HMP forms gels in the presence of a high concentration of cosolutes as sucrose and in acid media (pH < 3.5), while LMP forms gels in the presence of divalent cations, mainly Ca^2+^ [4,5]. Because of this ability, pectin has limited applications; these are explained by the inconsistencies in their chemical structure, which were determined by commercial extraction methods and the inherent source variability. To overcome these disadvantages, chemical or enzymatic modifications of these heteropolysaccharides were carried out. A wide variety of pectin derivatives were synthesized due to the abundance of hydroxyl and carboxyl groups located along the main skeleton of the polysaccharide or on its side chains consisting of neutral sugars. Thus, the quaternization of pectin with hydrophilic and/or hydrophobic reactants allows for obtaining soluble chemical compounds, with a controlled hydrophilic–hydrophobic balance and having several practical applications [16,17,18,19,20]. Most often, the quaternization reaction of polysaccharides was carried out using reagents that contained quaternary ammonium groups in their chemical structure. Two classic quaternization reagents are N-(3-chloro-2-hydroxypropyl)-N,N,N-trimethylammonium chloride and N-(glycidyl)-N,N,N-trimethyl ammonium chloride because they have low toxicity and good stability. So, a large number of polysaccharides, such as chitosan [21,22], konjac glucomannan [23], curdlan [24], sulfated xylan [25], pectin [16], methylan [26], K. terrigena polysaccharide [27], Ganoderma glucans [28], inulin [29], and potato starch [30] were quaternized using classical reagents. The reaction of the polysaccharides with the quaternization reagent was achieved in NaOH to obtain the conversion of the reagent into epoxide. But, the occurrence of a strong alkaline medium can impact the supramolecular structure of the polysaccharide or it can even destroy its chemical structure. Another protocol, followed for the of quats, is a one-pot procedure and consists of a reaction between the polysaccharide and a quaternization mixture containing a tertiary amine and ECH. This protocol is adaptable, as it enables the production of many polysaccharide derivatives simply by substituting the tertiary amine employed in the chemical reaction. Between the polysaccharides, dextran [31] and citrus pectin [32] were used for the synthesis of quaternary ammonium products by using the aforesaid method, and the resulting compounds showed varied applications. Thus, soluble quats based on dextran and citrus pectin displayed antipathogenic properties [31,32], whereas quaternized hydrogels based on dextran demonstrated adsorption capabilities for bile acid salts [33,34] and dyes [35,36].

The present work describes the synthesis of new QPAs produced via the reaction between PA and various reaction mixtures containing ECH and N,N-dimethyl alkylamine (alkyl = ethyl (Et), butyl (Bu), benzyl (Bz), octyl (Oct), dodecyl (Dod)) as tertiary amines, followed by an investigation of their structures, morphologies, and properties. The major aim of this study was the examination of PA and QPA antipathogenic activities due to PA, which has a recognized intrinsic antimicrobial activity [37]. These findings offer new perspectives into the physicochemical characteristics of QPAs and will enhance the promotion of novel antipathogenic materials derived from them.

## 2. Materials and Methods

### 2.1. Materials

Pectin from apple (PA) (poly-D-galacturonic acid methyl ester), epichlorohydrin (99%) (GC), N,N-dimethylbenzylamine (98%), N,N-dimethyloctylamine (95%), and NaOH (reagent grade, ≥98%) were bought from Sigma-Aldrich (Saint Louis, MO, USA), while N,N-dimethyl ethylamine (purum, ≥99%) (GC), N,N-dimethylbutylamine (purum, ≥98%) (GC), and N,N-dimethyldodecyl amine (≥90%) (GC) were received from Fluka (Buchs, Switzerland). HCl solution (37%) was acquired from the Chemical Company S.A., Iasi, Romania. *S. aureus* ATCC 25923, *E. coli* ATCC 25922, and *C. albicans* ATCC 10231 were acquired from the culture collection of the Department of Microbiology, Faculty of Pharmacy, “Gr. T. Popa” University of Medicine and Pharmacy, Iasi, Romania.

### 2.2. Chemical Modification

**Figure 1:**
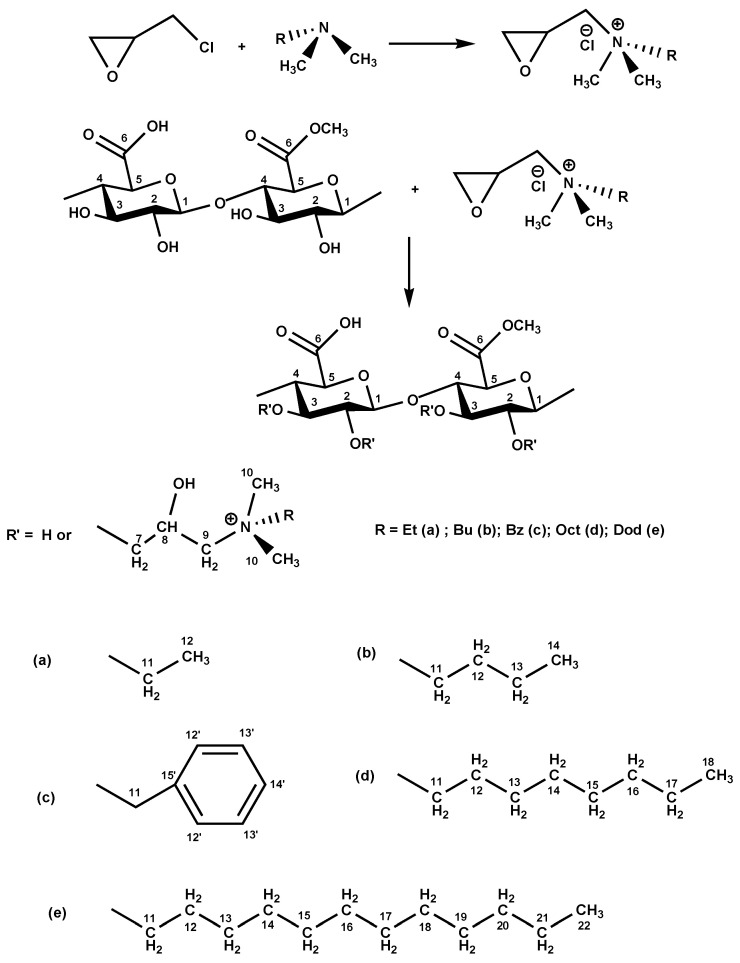
Chemical structure of QPA-RX (RX = Et44 (**a**), Bu38 (**b**), Bz27 (**c**), Oct35 (**d**), Dod14 (**e**)).

The synthesis of QPAs (Figure 1), achieved by pectin quaternization with a mixture of ECH and N,N-dimethylethylamine, is provided as a model.

Thus, PA (1 g, 0.0057 saccharide units) was dissolved in deionized water (20 mL) and stirred at r.t. until completely dissolved. The mixture of N,N-dimethylethylamine (8.1 mL, 0.075 mol) and ECH (5.4 mL, 0.069 mol) was dropwise added to the pectin solution, and the reaction system was kept under magnetic stirring at 70 °C for 6 h. The resulting solution was purified using a dialysis bag (12 kDa cut-off); first dialyzed against 0.1 N HCl (24 h) and then against deionized water until the conductivity of the dialysate was approximately equal to that of deionized water; and finally, freeze-dried.

Figure 1 shows the chemical structures of the PA derivatives, with their polymeric codes being QPA-RX, where R is the alkyl substituent [R = Et, Bu, Bz, Oct, Dod] and X = DS. Elemental analysis allowed for the determination of the nitrogen percent (N%), which afforded the calculation of the DS values of pectin derivatives. Equation (1) was used for the determination of DS (%), expressed in moles of pendant groups per 100 α-(l-4)-GA units:

(1) $$DS=\frac{176\times N\%}{\left[1400-(N\%\times M_{S})\right]}\times 100,mol/100\;GA,$$

where 176 is the molecular weight of the GA unit, N% is the nitrogen percent, and MS is the molecular weight of the pendant ammonium group.

The PA-based polymers coded as QPA-Et44, QPA-Bu38, QPA-Bz27, QPA-Oct35, and QPA-Dod14 had the chemical structures shown in Figure 1.

### 2.3. Analytical Methods

#### 2.3.1. Structural Characterization

A Bruker Avance NEO spectrometer (100.6 MHz for ^13^C nuclei) (Bruker, Rheinstetten, Germany) was used for the acquisition of the ^13^C NMR (D_2_O) spectra, and sodium 3-(trimethylsilyl)-[2,2,3,3-d4]-1-propionate (TSP) was employed as the internal standard. FTIR spectra were measured with a Bruker Vertex 70 spectrophotometer (Bruker, Ettlingen, Germany) using KBr pellets as matrix material. Elemental analysis (N%) was accomplished with the help of a Multi NC 3100 CLD analyzer (Analytic Jena, Jena, Germany).

#### 2.3.2. Determination of Ash and Moisture Contents

The ash content was determined by incinerating 0.2 g of PA in a furnace at 550 °C for 4 h, while the moisture percent was found by drying 0.2 g of the same samples in an air-circulating oven at 50 °C for 24 h. In both cases, the resulting residues were cooled and kept in a vacuum desiccator for several hours, followed by their weighing. Both the ash and moisture contents [38] were calculated with Equation (2):

(2) $$Ash/moisture\;(\%)=\frac{mass\;of\;the\;final\;residue}{mass\;of\;the\;initial\;pectin\;sample}\times 100$$

#### 2.3.3. Conductometric Titration

The indirect titration method was used for the quantification of both carboxyl and methyl-ester group contents of the PA [32]. The protocol for the calculation of the carboxylic groups content (mEq/g) was as followed: 10 mL of 0.1 M NaOH solution, added in excess in the aqueous solution obtained by the dissolution of 0.2 g PA in 40 mL of deionized water, was succeeded by a conductometric titration with a 0.1 N HCl solution. This back titration afforded the production of sodium pectate and the determination of the carboxyl group content of PA. The chart showing the variation of the conductivity vs. HCl volume had two inflexion points: the first related to the excess of NaOH and the second one to the neutralization of ^6^COONa groups. The HCl volume employed in the titration between the inflexion points ($V_{HCl}^{a}$) was utilized for the evaluation of pectin carboxylic groups ($c_{COOH}$) with the aid of Equation (3):

(3) $$c_{COOH}\;=\frac{V_{HCl}^{a}\times c_{HCl}^{a}}{m}\;mEq/g,$$

where $V_{HCl}^{a}$ (L) is the volume of the 0.1 N HCl solution used in the titration between the two inflexion points, $c_{HCl}^{a}$ (mol/L) is the molar concentration of the HCl solution, and *m* (g) is the quantity of the dry PA.

The protocol for the calculation of the total content of carboxyl and methyl-ester groups (mEq/g) was as follows: 10 mL of 0.25 M NaOH solution, added in excess in the aqueous solution obtained by the dissolution of 0.2 g PA in 40 mL of deionized water, was succeeded by the conductometric titration with a 0.25 N HCl solution. This reverse titration allowed for the hydrolization of ester groups to carboxyl groups and the calculation of the total amount of pectin carboxylic and ester groups (*c_COOH+COOCH_3__*) with the help of Equation (4):

(4) $$c_{COOH+{COOCH}_{3}}=\frac{V_{HCl}^{b}\times c_{HCl}^{b}}{m}\;mEq/g,$$

where $V_{HCl}^{b}$ (L) is the volume of the 0.25 N HCl solution employed in the titration between the two inflexion points of the graph, which represented the variation in the conductivity vs. the HCl volume, where the first inflexion point was connected to the excess of NaOH and the second one to the hydrolization of ester groups to carboxyl groups; $c_{HCl}^{b}$ (mol/L) is the molar concentration of the HCl solution; and *m* (g) is the mass of the dry PA.

#### 2.3.4. Viscometric Analysis

Viscometry was utilized for the calculation of the *M_v_* of PA by using the Houwink–Kuhn–Sakurada equation (Equation (5)) [39], while the intrinsic viscosity [η] was assessed with the aid of Solomon–Ciuta equation (Equation (6))[40]:

(5) $$[\eta ]=0.0174\times M_{v}^{0.84}(mL/g),$$

(6) $$[\eta ]=\frac{{\left[2\eta_{sp}-2ln(\eta_{rel})\right]}^{1/2}}{c},$$

Specific (*η_sp_*) and relative (*η_rel_*) viscosities were found out via a single measurement at a low pectin concentration (*c* = 25 × 10^−4^ g/mL) in deionized water by using an Ubbehlode viscometer (0a type) (SI Analytics, Mainz, Germany) introduced in a thermostated bath at 25 °C.

#### 2.3.5. DLS Technique

The hydrodynamic diameter (D_h_) and polymeric distribution of PA and its quats in deionized water were assessed by DLS using a Zetasizer model Nano ZS, with a red 633 nm He/Ne laser (Malvern Instruments, Worcestershire, UK) on a polymer solution (C_P_ = 10^−3^ g/mL) obtained by dissolving 0.01 g of polymer into 10 mL of deionized water. The backscattering detection system (detection at 173° to the incident beam) was used, and the results were the mean of threefold measurements for each sample (each measurement was obtained with 17 runs, 15 s run duration, automatic mode) at r.t. The D_h_ values were found by the intensity distribution using the method of cumulants.

#### 2.3.6. STEM

STEM analysis was conducted using a Verios G4 UC Scanning Electron Microscope (Thermo Scientific, Brno, Czech Republic) operating in STEM mode at 30 kV and equipped with a STEM 3+ detector. The samples were prepared following the TEM sample preparation protocol, which involved the deposition of the diluted sample (C_P_ = 10^−3^ g/mL) onto 400 mesh carbon-coated copper grids (TED PELLA) and allowing them to air-dry for 24 h in a dust-free environment at r.t. The resulting images were processed using ImageJ 1.48r software.

#### 2.3.7. Thermal Analysis

Thermogravimetric (TG) experiments were performed on a Discovery TGA 5500 (TA Instruments, New Castle, DE, USA). The samples with a weight of 6.4 mg were heated from r.t. up to 700 °C in a nitrogen atmosphere (25 mL/min) using a heating rate of 10 °C/min.

Differential scanning calorimetry (DSC) tests were performed using a Discovery DSC 250 (TA Instruments, New Castle, DE, USA) under a nitrogen atmosphere (50 mL/min). Samples with a mass of 6.5 mg were sealed in aluminum pans. The thermal behaviors of the samples were explored through heating—cooling—heating cycles between −100 °C and 180 °C using a heating/cooling rate of 10 °C/min. The temperatures were evaluated from the first heating cycle using TRIOS software 5.0.

#### 2.3.8. Wide-Angle X-Ray Scattering (WAXD)

WAXD data were acquired with an automated powder diffractometer Bruker D8 Avance (PW 1050) (Bruker, Kalsruhe, Germany) with Bragg–Brentano geometry. Bruker AXS computer software DIFFRAC-plus–Topaz 3.1 was employed to analyze and plot the data.

#### 2.3.9. Antimicrobial Activity

A Gram-positive bacterium (*S. aureus* ATCC 25923), Gram-negative bacterium (*E. coli* ATCC 25922), and pathogenic yeast (*C. albicans* ATCC 10231) were employed to test the antipathogenic activities of the PA and QPAs by using a disk diffusion method [41,42]. Mueller Hinton Agar (Oxoid) (Thermo Fisher Scientific, Vlaams Brabant, Belgium) and Mueller Hinton Agar Fungi (Biolab, Budapest, Hungary) were inoculated in Petri dishes with the suspensions of the above-mentioned microbes, followed by the introduction of sterile stainless-steel cylinders (5 mm internal diameter, 10 mm height) on an agar surface. After this, 0.1 mL of the polymer (C_P_ = 10^−2^ g/mL) was placed into cylinders. Petri dishes were held for 10 min. at r.t. to obtain a homogeneous partition of the PA and its derivatives, and subsequently, they were incubated at 35 °C for 24 h. Commercial discs that contained Ciprofloxacin (5 μg/disk) (Thermo Fisher Scientific, Vlaams Brabant, Belgium) and Nystatin (25 μg/disk) (Carl Roth, Karlsruhe, Germany) were utilized as antibacterial and antifungal reference drugs, respectively. After the incubation, the diameters of the inhibition zones were estimated in mm, including the disc size.

## 3. Results and Discussion

### 3.1. Ash and Moisture Contents of PA

The purity and quality of the PA were tested by determining the values of the ash and moisture percentages, respectively, according to the protocols described in Section 2.3.2. Thus, the ash content of neat PA was 1.94%, which was close to that detected for the banana peel pectin (1.43–3.46%) [43]. It is known that the purity of materials is associated with their ash analysis. So, the low value found for the PA ash content (1.94%) indicated a high purity of the polysaccharide. The value of the moisture percent determined for the PA, 5.4%, was close to that of soy hull pectin (6–7%) [44], banana peel pectin (4.54–7.92%) [43], and pectin from wild fruits of Odisha (3.84–7.69%) [45]. The low level of moisture content found for pristine pectin is an indicator of the quality because a reduced wetness level determines a high ability to inhibit the development of microorganisms and pectinase enzymes [46]. The PA sample, with a verified purity and quality, was quaternized according to the procedure mentioned in Section 3.4.

### 3.2. The Methoxylation Degree of Native Pectin

The DM value of PA was determined by using back conductometric titration with HCl of the mixture consisting of dissolved PA and NaOH solution, which was added in excess. The determination of the content of pectin carboxylic groups (*c_COOH_*) required the employment of 0.1 N solutions of NaOH and HCl, while for the calculation of the total content of carboxyl and methyl-ester groups (*c_COOH+COOCH_3__*), 0.25 N solutions of NaOH and HCl were utilized. The amount of pectin methyl-ester groups (*c_COOCH_3__*) (1.898 mEq/g) was determined by subtracting the content of pectin carboxylic groups (*c_COOH_*) (2.811 mEq/g) from the total amount of carboxyl and methyl-ester groups (*c_COOH+COOCH_3__*) (4.703 mEq/g). DM value found for PA and calculated with Equation (7), taking into account the above-mentioned values, gave 40.35%, which means that the PA was a low-methoxyl pectin (DM < 50%) [3].

(7) $$DM\;(\%)=\frac{c_{COOH+{COOCH}_{3}}-c_{COOH}}{c_{COOH+{COOCH}_{3}}}\times 100$$

### 3.3. Viscosity Measurements for Unmodified Pectin

The M_v_ of PA could be found using capillary viscometry technique [24]. The M_v_ was computed using the Houwink–Kuhn–Sakurada equation (Equation (5)), while [η] was established from a single measurement at a low polymeric concentration (C_P_ = 25 × 10^−4^ g/mL) by using the Solomon–Ciuta equation (Equation (4)). The values showed by the PA for its [η] (393.4 mL/g) and M_v_ (1.53 × 10^5^) are in good agreement with those found for other pectin isolated from citrus fruit peels [32,39] or orange and grapefruit peels [47].

### 3.4. Synthesis of Quaternary Ammonium Derivatives of Pectin

PA and different quaternization mixtures (N,N-dimethylalkylamine + ECH) reacted under magnetic stirring at 70 °C for 6 h by a one-pot reaction to generate well-controlled polymeric structures with quaternary ammonium pendant groups statistically distributed along the pectin chain (Figure 1). The tertiary amine (N,N-dimethyl-N-alkyl amine) (where alkyl = Et, Bu, Bz, Oct, or Dod) reacted with ECH to generate the quaternary ammonium reagent in situ, which subsequently attacked the PA. A slight excess of tertiary amine served as the catalyst, which enhanced the DS values of the obtained polymers. By simply altering the tertiary amine, a variety of quaternized pectins with distinct properties could be produced. QPAs are white solid compounds, and their DS values were calculated by knowing the N% (elemental analysis) with the help of Equation (1) (Table 1). The DS and yield (η%) of the amination reaction decreased for pectin derivatives with the augmentation of the alkyl length of N,N-dimethylalkylamine due to the reduction in tertiary amine water solubility, as well as the steric hindrance produced in quaternization reaction in the case of voluminous alkyl substituents (Bz, Oct, Dod) of the amine (Table 1). Furthermore, the solubility of native pectin in water, given by its DM value, directly influenced the DS of the obtained quats. So, the QPAs acquired using the same molar ratio of pectin/quaternization mixture and the same reaction conditions as those used for the quaternization of citrus pectin showed higher DS values in comparison with the quats obtained from citrus pectin due to the increased solubility in the water of PA (DM < 50%) compared with that of citrus pectin (DM > 50%) [32].

### 3.5. FTIR Analysis

**Figure 2:**
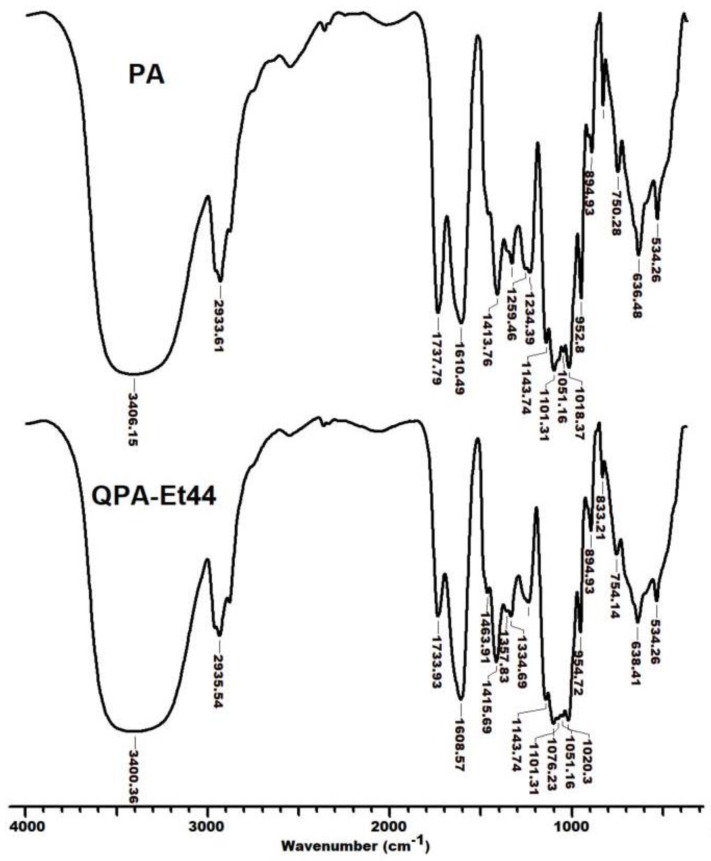
FTIR spectra of PA and QPA-Et44.

The FTIR studies proved the production of quats based on apple pectin (Figure 2) and the analysis of the FTIR spectra of the PA and QPAs showed the existence of common bands (i) and specific bands (ii) (Table 2). (i) A wide band, which belonged to the stretching vibrations of O–H groups, could be observed in the FTIR spectra of both the PA (3441 cm^−1^) and QPAs (3427–3435 cm^−1^). This band is usually wide-ranging due to the existence of intermolecular hydrogen bonds between the numerous -OH groups present in the chemical structure of pectin and its derivatives. A narrow band characteristic of the stretching vibrations of C–H groups had an enhanced area for the aminated polymers (2923–2925 cm^−1^) compared with that of the PA (2934 cm^−1^) as a result of the supplementary –CH_2_– groups of the alkyl substituent R (Figure 1).

Narrow peaks, ascribed to the stretching vibrations of C=O (ester) and ^6^COO^−^ groups occurred mutually in the PA (C=O_ester_: 1749 cm^−1^; ^6^COO^−^: 1643 cm^−1^) and QPA (C=O_ester_: 1735–1741 cm^−1^; ^6^COO^−^: 1611–1617 cm^−1^) spectra, with the mention that for the C=O (ester) groups, the peak areas were smaller in the cases of the QPAs in comparison with that of the PA, and the ^6^COO^−^ groups peak areas of the QPAs were larger when compared with those of the PA due to the partial hydrolysis of the ester groups during the synthesis, which was caused by the excess amine used in the quaternization. The FTIR spectra of the PA and its quats showed the existence of stretching vibration bands characteristic of C–O (glycosidic backbone) (PA: 1105 cm^−1^; QPAs: 1099–1105 cm^−1^) and C–C (pyranoid ring) (PA: 1017 cm^−1^; QPAs: 1016–1019 cm^−1^) [48]. (ii) Peaks that corresponded to the stretching vibrations of –CH_3_ groups bonded to the quaternary nitrogen of polymeric side chains (1465–1481 cm^−1^), as well as peaks assigned to the stretching vibrations of the C–N group (1414–1417 cm^−1^), could be observed in all the FTIR spectra of the QPAs, which confirmed their production. The existence of either rocking bands for –(CH_2_)_n_– (n > 5) in the spectra of amphiphilic derivatives with octyl (723 cm^−1^) and dodecyl (720 cm^−1^) substituents [49] or bands specific to in-plane (ip) C–H bending (1045 cm^−1^) and out of-plane (oop) C–H bending (738 cm^−1^) that occurred in the benzyl-containing polymer spectrum also revealed the successful amination of PA. The values of FTIR peaks showed by the QPAs were in good agreement with those seen for other quaternized polysaccharides, such as konjac glucomannan [23], curdlan [24], sulfated xylan [25], methylan [26], Ganoderma glucans [28], citrus pectin [32], and hawthorn pectin [19].

### 3.6. ^13^C NMR Spectroscopy

**Figure 3:**
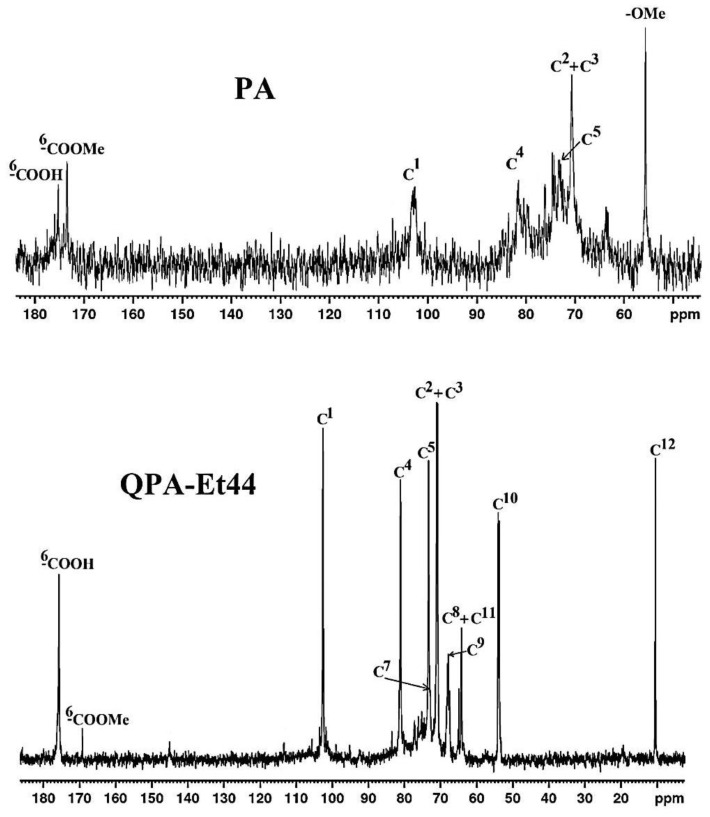
^13^C NMR (D_2_O) spectra of PA and QPA-Et44.

The NMR characterization of the QPAs structures, which was conducted by using ^13^C NMR due to a limited separation of the peaks in the ^1^H NMR spectra, demonstrated the effective amination of the native pectin. The ^13^C NMR spectra of the PA and one of its derivatives, QPA-Et44, chosen as an example, are shown in Figure 3, and the chemical shifts for all polymers are revealed in Table S1.

The carbon atoms of QPAs were numbered according to Figure 1. In the ^13^C NMR spectrum of PA, the signals for C^6^ could be observed at 176.9 and 173.65 ppm, which corresponded to the ^6^COOH and ^6^COOMe units, respectively. Also, the signals for C^1^, C^4^, and C^5^ were found at 103.32, 81.45, and 73.67 ppm, respectively. The chemical shifts of the carbon atoms of PA were in accordance with the values reported in the literature for other pure pectins [50]. The peak, which corresponded to the carbon atoms of the methyl group from ^6^C-methyl-esterified units, coded as –OMe, was detected at 55.9 ppm, while the signals for C^2^ and C^3^ were overlapped and occurred at 70.56 ppm. The ^13^C NMR spectra of QPA-Et44 (Figure 3), used as an example, demonstrated the functionalization of the PA with quaternary ammonium groups as polymeric side chains. So, the ^13^C NMR spectrum of QPA-Et44 displayed peaks at 169.03 ppm and 176.21 ppm, which corresponded to the ^6^C from the PA unit, which were methyl-esterified or un-esterified, respectively. The signals corresponding to the carbon atoms from the quaternary ammonium side chains also occurred in the ^13^C NMR spectrum of QPA-Et44, specifically C^12^ (10.53 ppm), C^11^ (64.76 ppm), C^10^ (54.13 ppm), C^9^ (68.13 ppm), C^8^ (64.31 ppm), and C^7^ (72.75 ppm). There were mutual peaks with the ^13^C NMR spectrum of the PA, which occurred in the QPA-Et44 spectrum, as follows: C^1^ (103.44 ppm), C^2^ (71.07 ppm), C^3^ (70.82 ppm), C^4^ (80.94 ppm), and C^5^ (72.98 ppm). The presence of all previously found peaks in the ^13^C NMR spectra of QPAs indicates the successful attachment of quaternary ammonium groups on the pectin backbone. Additionally, the ^13^C NMR chemical shift values observed for the quats of PA were consistent with those found for the quaternary ammonium compounds of different polysaccharides, like cellulose [51], starch [52], dextran [31], curdlan [24], sulfated xylan [25], Ganoderma glucans [28], citrus pectin [32], and hawthorn pectin [19].

### 3.7. DLS Technique

**Figure 4:**
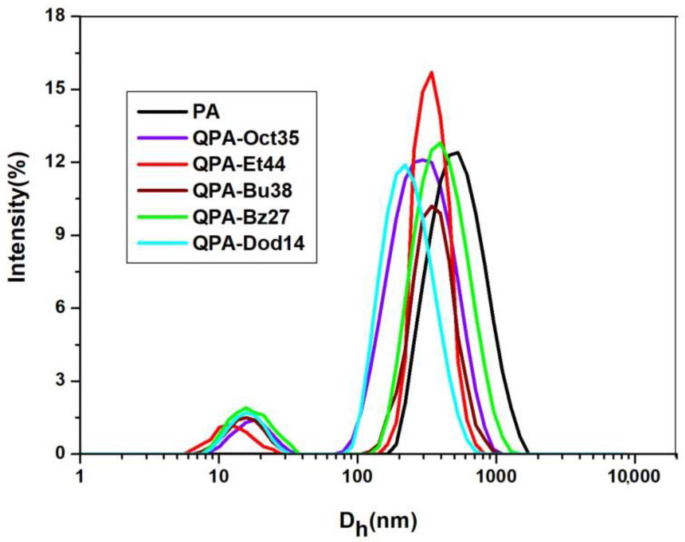
Size distribution of PA and QPA chains in aqueous solution.

DLS is a widely used method for determining the D_h_ of macromolecules in an aqueous solution, where the values are influenced by the polymeric average particle size and the size distribution. This technique offers the benefits of being quick, non-invasive, and needing low concentrations (C_P_ = 10^−3^ g/mL for PA and QPAs). A unimodal distribution can be seen by looking at the PA histogram with a D_h_ = 490 nm due to the stronger repulsive electrostatic forces between charges of the same sign (^6^COO^−^) compared with the attractive hydrogen bonds between the pectin OH groups (Figure 4, Table 1).

A bimodal distribution can be seen in the QPA histograms (Figure 4, Table 1), but the D_h_ and relative proportions of the two populations were influenced by the DS value and pendant group hydrophobicity of the pectin-based quats. The QPA aqueous solutions contained a main population with a D_h_ of about 185–334 nm and a low percentage of small particles (D_h_ = 20–30 nm). The analysis of the particle volume size distribution (data not displayed) revealed a significant dominance of the population with higher D_h_ values (185–334 nm). As a result, Table 1 includes only the sizes related to the main population. The D_h_ values of the QPAs were lower than those of the PA due to the partial de-esterification and depolymerization [53] of the pectin derivatives during the synthesis and also due to the attractive forces (the electrostatic and hydrogen bonds for all the QPAs and the hydrophobic ones for the amphiphilic derivatives) between the polymeric chains. The lowest values of D_h_ were recorded in the cases of the QPAs with Oct- and Dod-containing pendant groups due the hydrophobic interactions between the polymeric side chains, along with the electrostatic and hydrogen bonds, which favored the aggregation process. The D_h_ values found for the PA and QPAs were in line with those determined for other pectins, such as citrus pectin [32] and sugar beet pectin [54].

### 3.8. STEM Analysis

**Figure 5:**
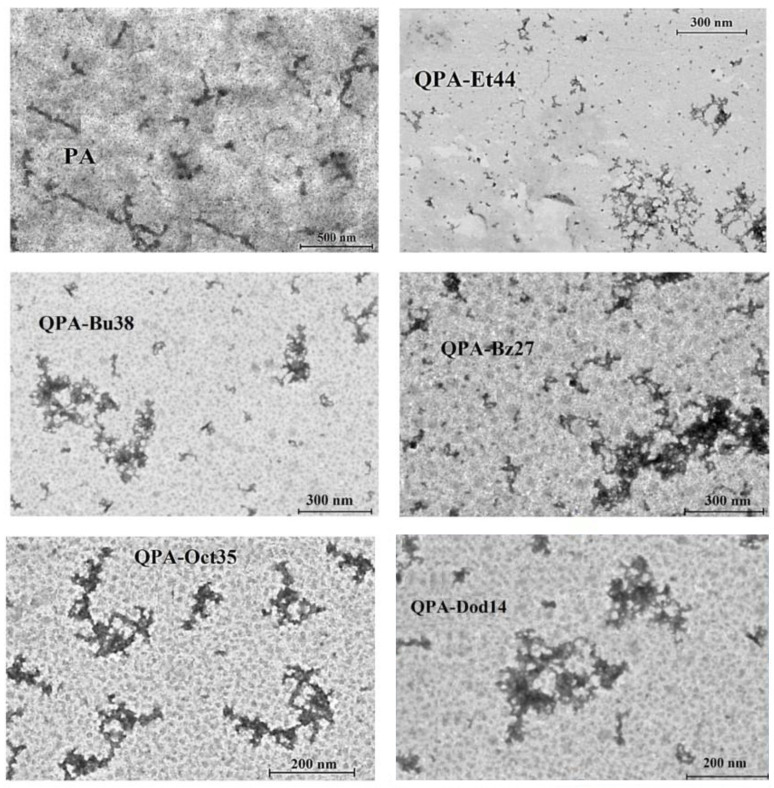
STEM images of PA and QPAs.

Scanning transmission electron microscopy operates in both transmission and reflection electron imaging modes, employing a focused scanning beam of incident electrons. It is regarded as a highly effective tool for characterizing materials due to its distortion correctors that significantly enhance its resolution. Figure 5 shows the STEM images of the parent polymer and its derivatives.

The microscopy images of the PA indicate that most pectin chains were in an extended rod-like conformation with lengths up to 450 nm due to the electrostatic repulsions between the ^6^COO^−^ groups. The occurrence of a few polymeric aggregates for apple pectin with lengths that could reach up till 1 μm could be explained thanks to attractive interactions (hydrogen bonds) between the PA chains. The literature shows the formation of PA aggregates, even at high dilutions, which proves that they are not simply the outcome of overlapping or entangling polymer chains [55]. The STEM analysis for the QPAs showed a few single polymeric chains, along with numerous pectin aggregates, with sizes that ranged from hundreds of nanometers to one micron. The dimensions of the polymeric aggregates formed by the QPAs were smaller compared with those of the native pectin due to the partial de-esterification and depolymerization reaction [53] during the synthesis and the attractive forces between the polymeric chains: electrostatic and hydrogen bonds (specific to all QPAs) and hydrophobic forces (characteristic of amphiphilic derivatives). The most bulky aggregates were those formed by QPA-Bz27 due to the voluminous pendant benzyl groups. The amphiphilic derivatives (QPA-Oct35 and QPA-Dod14) had smaller sizes of polymeric aggregates compared with those formed by the hydrophilic derivatives (QPA-Et44 and QPA-Bu38) due to hydrophobic interactions between lipophilic side chains.

### 3.9. Thermal Measurements

**Figure 6:**
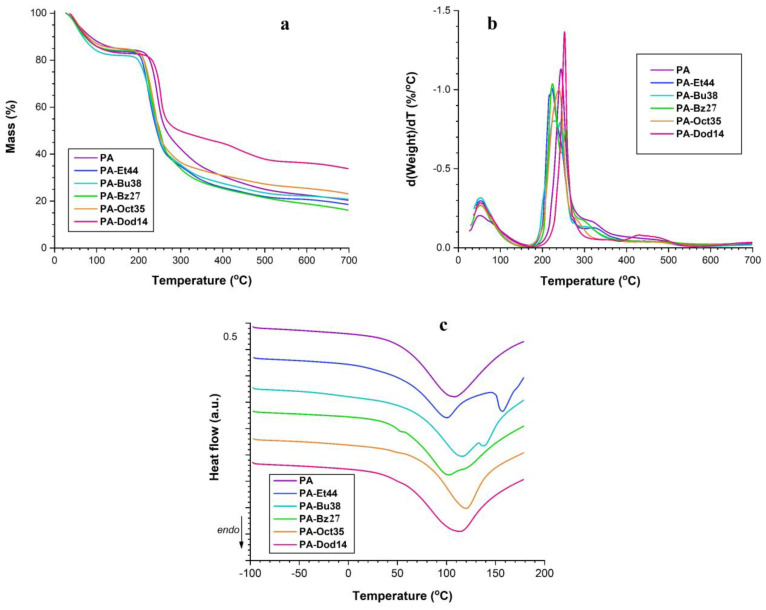
Thermal analysis of PA and QPAs: TG (**a**), DTG (**b**), and DSC (**c**).

The thermal behavior of pure pectin and its derivatives was studied by TGA and DSC techniques, and the obtained results are represented graphically in Figure 6 and summarized in Table S2. Thermal degradation of the PA and QPAs in a N_2_ atmosphere, which contained four decomposition stages (Figure 6a,b), was a complex set of reactions related to water evaporation (dehydration) and thermal degradation (pyrolytic decomposition and decarboxylation), followed by char gasification [56,57]. The high initial mass loss (14–17%), detected at temperatures below 150 °C, could be explained by the high hydrophilicity of pectin due to the occurrence of numerous hydroxylic and carboxylic groups in its chemical structure. Also, the partial de-esterification and de-polymerization reactions, which occurred during the quaternization of pectin, determined an enhanced affinity for water of the quats due to the augmentation of their content in ^6^COOH groups (by de-esterification) and OH groups (by depolymerization). This initial mass loss was attributed to the release of water, which was either adsorbed on the polymer surface and interacted solely with other water molecules or retained inside the spaces between the polysaccharide backbone chains by hydrogen bonds with pectin hydroxyl groups and/or ion-dipole bonds with quaternary ammonium pendant groups [58]. The DSC analysis up to 200 °C helped to detect two types of water for the PA and QPAs (Figure 6c). The existence of only one endothermic process in the DSC curve (at T^1^) (Table S2) indicates the superficial retention of water molecules (on polymeric surface), while the occurrence of two endothermic peaks (at T^1^ and T^2^) (Table S2) suggests the presence of two types of water. QPA-Et44 and QPA-Bu38 showed two distinct endothermic peaks up to 200 °C due to their short pendant alkyl substituent, which allowed the water molecules to be retained more easily in the depth of polymeric chains by hydrogen and ion-dipole bonds. The slight shift in the T^1^ and T^2^ values for the different QPAs was related to the different polymeric water contents and conformations [59]. The second DSC heating scan also confirmed this hypothesis: the DSC peaks disappeared after cooling and rescanning. The second and third decomposition stages (39–59% mass loss), which occurred between 200 °C and 400 °C, were characterized by multiple and simultaneous processes correlated with the PA’s and QPAs’ complex composition and structure. So, in these two stages, the polymeric chains experienced significant thermal degradation first; followed by decarboxylation of the ^6^COOH groups (PA and QPAs) and GA ring (PA and QPAs); and finally, the liberation of various gaseous products with the generation of a solid char [19]. It is difficult to precisely state at what stage of the thermal decomposition the cleavage of the pendant groups will take place and what compounds will be released since their degradation is part of a broader decomposition process. Moreover, TGA can only indicate the presence of certain types of volatilization products, but it does not provide direct chemical information. QPA-Bz27 exhibited a more complex thermal behavior, highlighted by the presence of a secondary peak in the second stage of the degradation process at a temperature higher than 250 °C due to the occurrence of aromatic rings in its pendant groups [60]. The fourth region (400–600 °C) showed a slow mass loss (5–10%) determined by the pyrolytic degradation of char with the formation of a partially destroyed and densely packed solid char that included polyaromatic structures that contained aliphatic and ketonic groups [61].

The QPAs were more sensitive to thermal degradation compared with the PA due to the occurrence of alkyl/aryl substituents in the polymeric pendant groups, which may disrupt the hydrogen bonds and intermolecular interactions between the pectin molecules and make them more susceptible to thermal decomposition. The existence of benzyl substituents in pendant groups stabilized the chemical structure through electronic and steric effects and made the QPBz27 more thermally stable. Unlike the short alkyl substituents (Et, Bu) of the pendant groups, the Dod substituents induced the greatest thermal stability between the QPAs due to their highest hydrophobicity, which improved the molecular packing of the polysaccharide chains, reduced the mobility, and increased the resistance to thermal degradation. So, the temperature at which the degradation rate was at a maximum (T_max_) (Table S2) was augmented for the QPAs with the increase in the length of the alkyl substituent (Figure 1). Also, the DTG curve (Figure 6b) representing the first derivative of the weight of samples versus temperature indicates that the presence of long alkyl substituents or aromatic nuclei in the polymeric pendant groups decreased the decomposition rate. The mass residues of the QPAOct35 and QPADod14 were bigger than those of the PA and the rest of the quats due to the longer lengths of their pendant groups. The results of the TGA/DTG analysis proved a decrease in the thermal stability of pectin after its quaternization. Moreover, the data obtained were in accordance with those acquired by thermal stability studies conducted with quats based on different polysaccharides, such as curdlan [62], chitosan [63], or agar [64]. The pectin degradation backbone is usually shown in a DSC curve by an exothermic peak, which starts at temperatures higher than 200 °C [19]. This peak is not present in Figure 6c because the DSC analysis was performed only up to the temperature at which the PA and QPAs started to decompose in order to avoid the degradation of the samples in the DSC cell.

### 3.10. WAXD Technique

**Figure 7:**
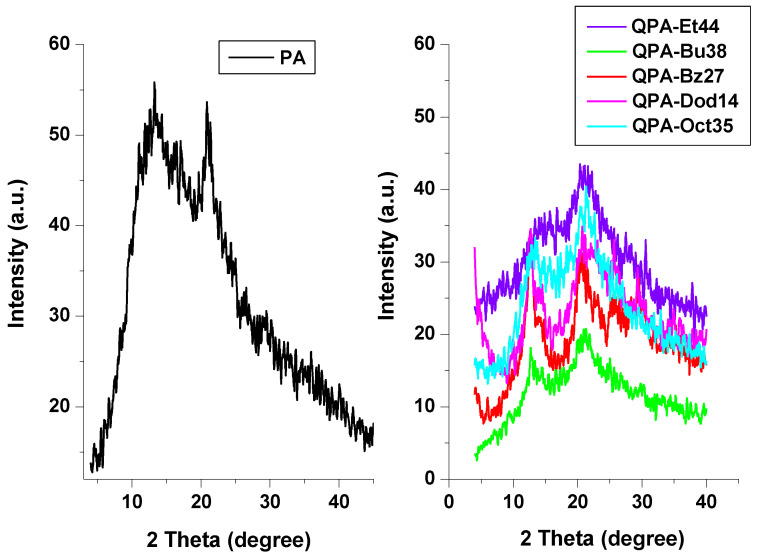
WAXD spectra of PA and its derivatives.

The WAXD patterns of the PA and QPAs were examined in order to investigate the polymeric structural properties. So, the PA showed two broad typical peaks at 13.2° and 20.9° (Figure 7). The peak at 13.2° moved to about 12.7° for the quaternized compounds, while the peak at 20.9° was not altered. In addition, both peaks’ intensities decreased for the QPAs, which proved a decrease in the pectin’s crystalline structure during the quaternization process. The crystallinity, determined by the level of structural order within the polymer chains, decreased after the quaternization reaction due to the destruction of the intramolecular hydrogen bonds as a result of the grafting of quaternary ammonium pendant groups onto the OH groups of the pectin skeleton [19]. Similar drops in the crystallinity caused by the same grafting reaction onto the various polysaccharide backbones were reported for the quaternized derivatives of agar [64], curdlan [62], chitin [65], and pectin [19]. The decrease in the crystallinity observed for the QPAs was in good accordance with the TGA and DSC studies (Section 3.7), which indicate the reduction in thermal stability of the pectin derivatives in comparison with the pure polysaccharide.

Between the quaternized derivatives of pectin, those with a long alkyl substituent or aromatic ring in pendant groups (QPA-Bz27, QPA-Oct35 and QPA-Dod14) showed the higher crystallinity (Figure 7), which agreed with data obtained for the thermal degradation studies (Section 3.9), which indicates the greatest thermal stability for the above-mentioned polymers.

### 3.11. Antipathogenic Activity

The antibacterial and antifungal activities of the PA and QPAs were investigated because PA is known to have antimicrobial activity per se [37]. A pathogenic yeast (*C. albicans* ATCC 10231), Gram-negative bacterium (*E. coli* ATCC 25922), and Gram-positive bacterium (*S. aureus* ATCC 25923) were used for this study of the antipathogenic activity of pectin and its derivatives, and the sizes of the inhibition zones are displayed in Table 3. The antibacterial and antifungal properties of the PA and QPAs were tested using Ciprofloxacin and Nystatin, respectively, as positive controls. For C_P_ = 10^−2^ g/mL, all polymers showed antimicrobial efficacy against the pathogenic fungus and Gram-positive bacterium, but not against the Gram-negative bacterium. This behavior can be explained by taking into account the fact that Gram-positive bacteria have their peptidoglycan layer located outside the plasma membrane, which greatly facilitates the crossing of various biocides, whilst for Gram-negative bacteria, the peptidoglycan layer, occurring between the inner and outer cell membranes, makes the passive diffusion of the biocide very difficult [66,67]. Although their exact mechanism of action is still unknown, prior research demonstrated that pristine pectins are antimicrobials with wide-range spectra [37,68,69]. As a result, the antimicrobial activity of the PA was tested and the results demonstrated its positive activity against both *S. aureus* and *C. albicans*. The biocidal mechanism of action exercised by polymers with quaternary ammonium groups consisted of their adsorption and penetration of the microbial cell wall, with the disruption of the phospholipids of the cytoplasmic membranes of microbes, followed by the leakage of intracellular materials and degradation of proteins and nucleic acids, which finally led to cell lysis and the death of the pathogen. Polymeric derivatives with medium-length alkyl side chains (Bz and Oct) exhibited a higher antimicrobial activity against pathogens due to the presence of both hydrophobic and ionic interactions between them and the phospholipid bilayer of the microbes’ cell membrane. Polymers with short (Et, Bu) and long (Dod) alkyl substituent groups revealed diminished antimicrobial activities due to the lack of lipophilic interactions in the first case and the difficulty of the polymer to pervade the microbial cell membrane in the second case. Ciprofloxacin and Nystatin, used as positive controls, were more active than the QPAs. Table 3 contains the values of inhibition (%), which were calculated via multiplying the ratio between the value of the inhibition diameter (mm) of each polymer and the inhibition diameter (mm) of its reference drug (for antibacterial activity—Ciprofloxacin and for antifungal activity—Nystatin) by 100. The antibacterial and antifungal activities could also be seen for other quaternized derivatives of various polysaccharides, such as chitosan [70,71,72], konjac glucomannan [23], cashew gum [73], angico gum [74], the extracellular polysaccharide produced by K. terrigena [27], citrus pectin [16,32], and hawthorn pectin [19].

## 4. Conclusions

Novel quats derived from PA were effectively obtained by a one-pot procedure that consisted of a reaction between the polysaccharide and different quaternization mixtures composed of ECH and a tertiary amine. The DS values of the QPAs decreased with the augmentation of the hydrophobicity of the tertiary amine used in the synthesis. The formation of the pectin derivatives was confirmed by FTIR and ^13^C NMR techniques. Unimodal (PA) and bimodal (QPAs) distributions of polymeric aggregates were observed using DLS studies. STEM analysis revealed the occurrence of single polymeric chains and aggregates for the PA and its derivatives. Both in-solution (DLS) and in-solid-state (STEM) analyses showed that the smallest dimensions of the aggregates could be seen for the hydrophobically modified polymers. The thermal stability and WAXD analyses showed that the QPAs exhibited reduced thermal stability and crystallinity compared with those of the PA. This study of the antipathogenic activity confirmed positive results for the PA, already known from the literature, and indicated the QPAs as new antibacterial and antifungal agents against the *S. aureus* ATCC 25923 bacterium and *C. albicans* ATCC 10231 pathogenic yeast with improved activity compared with that of their parent pectins. Studies on the synthesis of novel quaternized polymers based on other pectins with increased DS values (by the augmentation of the molar ratio between pectin and quaternization mixture) compared with those reported in this paper, as well as their antipathogenic and antioxidant activities, will be the subjects of an upcoming work.

## Figures and Tables

**Table 1:** Color, N%, η%, and D_h_ values of PA and QPAs.

Polymer Code	PA	QPA-Et44	QPA-Bu38	QPA-Bz27	QPA-Oct35	QPA-Dod14
Polymeric color	Cream	White	White	White	White	White
N%	-	2.64	2.25	1.65	1.95	0.92
η (%)	-	62	51	44	25	15
D_h_ (nm)	490	245	287	334	227	185

**Table 2:** FTIR data of PA and QPAs.

Polymer Code	Type of Bond									
	O–H	C–H _(Aliphatic)_	C=O _(Ester)_	C=O_acid_ _(Ionic Form)_	CH_3_–N^+^	CH_3_–(CH_2_)_n_–N^+^	C–N	C–O _(glycosidic Ring)_	C–C ^(Pyranoid Ring)^	C–H _(Aromatic)_
PA	3441	2934	1749	1643	-	-	-	1105	1017	-
QPA-Et44	3427	2924	1737	1612	1485	-	1417	1103	1018	-
QPA-But38	3435	2924	1738	1614	1465	-	1415	1099	1019	-
QPA-Bz27	3428	2923	1741	1617	1481	-	1417	1102	1017	1045/738
QPA-Oct35	3428	2926	1740	1614	1468	723	1416	1103	1016	-
QPA-Dod14	3428	2923	1741	1617	1481	720	1414	1102	1019	-

**Table 3:** Antipathogenic activity of PA and QPAs.

Polymeric Code	Value of Inhibition (%)	
	*S. aureus* ATCC 25923	*C. albicans* ATCC 10231
PA	33%	48%
QPA-Et44	38%	64%
QPA-Bu38	47%	52%
QPA-Bz27	56%	68%
QPA-Oct37	62%	72%
QPA-Dod14	42%	48%
Ciprofloxacin	100%	-
Nystatin	-	100%

## Data Availability

The original contributions presented in this study are included in the article/Supplementary Material. Further inquiries can be directed to the corresponding author.

## Supplementary Materials

The following supporting information can be downloaded from https://www.mdpi.com/article/10.3390/polym16233352/s1, Table S1. ^13^C NMR chemical shifts of PA and QPAs; Table S2. Thermal parameters of PA and QPA.
