# Supplementary material for: Novel Quaternary Ammonium Derivatives Based on Apple Pectin
Source: Polymers (Basel). 2024 Nov 29;16(23):3352. doi: 10.3390/polym16233352 (PMC11644282; doi:10.3390/polym16233352)
Supplement: Supplementary file 1 [file polymers-16-03352-s001.zip › polymers-3326865-supplementary.pdf]

Supplementary Material

Novel quaternary ammonium derivatives based on apple pectin

Magdalena-Cristina Stanciu[1,*], Daniela Ionita[1], Daniel Tîmpu[1], Irina Popescu[1], Dana Mihaela Suflet[1], Florica Doroftei[1], Cristina G. Tuchilus[2]

[1]"Petru Poni" Institute of Macromolecular Chemistry, 41A, Gr. Ghica-Voda Alley, Iasi, 700487, Romania; cstanciu@icmpp.ro (M.-C.S.); ionita.daniela@icmpp.ro (I.D.); dtimpu@icmpp.ro (D.T.); ipopescu@icmpp.ro (I.P.); dsuflet@icmpp.ro (D.M.S.); florica.doroftei@icmpp.ro (F.D.)

[2]"Grigore T. Popa" University of Medicine and Pharmacy, Faculty of Medicine, 16, University Street, Iasi, 700115 Romania; ctuchilus@yahoo.com (C.G.T.)

* Correspondence: cstanciu@icmpp.ro

Table S1. ^{13}C NMR chemical shifts of PA and QPAs

Type of atom	δ (ppm) (D_2O, 100 MHz)					
	**PA**	**QPA-Et44**	**QPA-Bu38**	**QPA-Bz27**	**QPA-Oct35**	**QPA-Dod14**
C^{1}	103.32	103.44	102.63	102.87	102.79	101.86
C^{2}	70.56	71.07	71.11	70.89	70.86	71.53
C^{3}	70.56	70.82	70.83	70.68	70.48	70.89
C^{4}	81.45	80.94	80.99	81.00	81.07	81.57
C^{5}	73.67	72.98	73.56	73.07	73.07	74.08
C^{6} (COOH)	176.9	176.21	176.11	174.98	175.41	178.43
C^{6} (COOMe)	173.65	169.03	169.63	168.48	168.53	172.06
C^{7}	-	72.75	72.80	72.53	72.63	72.98
C^{8}	-	64.31	68.15	68.14	67.51	68.55
C^{9}	-	68.13	68.93	72.26	68.76	71.36
C^{10}	-	54.13	54.22	53.61	54.42	54.47
C^{11}	-	64.76	68.56	71.24	68.24	68.85
C^{12}	-	10.53	20.52	-	24.63	24.87
$C^{12'}$	-	-	-	135.77	-	
C^{13}	-	-	26.5	-	28.3	28.33
$C^{13'}$	-	-	-	131.54	-	
C^{14}	-	-	15.4	-	30.4	30.85-31.48
$C^{14'}$	-	-	-	133.51	-	
C^{15}	-	-	-	-	33.64	33.98
$C^{15'}$	-	-	-	129.74	-	
C^{16}	-	-	-	-	24.4	24.5
C^{17}	-	-	-	-	30.4	30.85-31.48
C^{18}	-	-	-	-	16.15	30.85-31.48
C^{19}	-	-	-	-	-	30.85-31.48
C^{20}	-	-	-	-	-	30.85-31.48
C^{21}	-	-	-	-	-	30.85-31.48
C^{22}	-	-	-	-	-	16.29
-OMe	55.9	53.2	53.9	53.04	54.23	54.33

Table S2. Thermal parameters of PA and QPA

Sample	TGA				DSC	
	Stages (°C)	T_{max} (°C)	Δw (%)	Residue (%)	T^1 (°C)	T^2 (°C)
**PA**	**Stage I**: 41.68 – 100.7	45.97	15.50	20.58	108.3	–
	**Stage II**: 228 – 257.5	244.2	42.23			
	**Stage III**: 306 – 342.4	293.8	10.85			
	**Stage IV**: 409.5 – 477.5	429.1	9.54			
**QPA-Et44**	**Stage I**: 49.17 – 87.63	52.25	15.41	18.79	100.9	157
	**Stage II**: 206.3 – 248.3	222.9	48.50			
	**Stage III**: 313.9 – 357.4	320.5	9.42			
	**Stage IV:** 434.8 – 507.9	441.6	4.22			
**QPA-Bu38**	**Stage I**: 47.08 – 85.84	52.62	16.84	20.95	116.4	138.3
	**Stage II**: 205.3 – 254.5	226.9	44.72			
	**Stage III**: 303.8 – 337.4	311	8			
	**Stage IV**: 415.6 – 506.6	455.5	4.91			
**QPA-Bz27**	**Stage I**: 42.3 – 88.6	52.16	15.74	16.4	103.1	120.4
	**Stage II**: 210.8 – 231.5;	224	30.75			
	251.2 – 262.7	255.1	16.55			
	**Stage III:** 286 – 326	292.9	11.60			
	**Stage IV:** 412.1 – 493.9	473.3	6.93			
**QPA-Oct35**	**Stage I**: 50.2 – 90.3	51.63	14.44	23.37	120.3	–
	**Stage II**: 213.2 – 250.2	239.3	43.05			
	**Stage III**: 274.9 – 296.6	283.5	6.46			
	**Stage IV:** 348.6 – 456	449.4	7.87			
**QPA-Dod14**	**Stage I**: 47.18 – 90.79	51.81	16.78	34.08	114.1	–
	**Stage II**: 240.6 – 253.1	252.6	29.37			
	**Stage III**: 269.3 – 282.6	276.7	8.17			
	**Stage IV:** 409.1 – 492.9	430.2	9.75			

T_{max} – temperature at which the degradation rate is maximum; Δw – mass loss
